# Antibodies vs. aptamers in cancer diagnosis and therapy: a comparative analysis of clinical applications, mechanisms, and future directions

**DOI:** 10.1097/MS9.0000000000005524

**Published:** 2026-10-26

**Authors:** Shreya Singh Beniwal, Barjola Hodaj, Akash Rawat, Yujin Jeong, Daniela Castro Calderón, Maria Fernanda Navarro Salcedo, Rafael Everton Assunção Ribeiro da Costa, Het Tushar Jani, Alyanna Cabe Cacas, Chimuka Mwaanga, Aarushi Mishra

**Affiliations:** a Lady Hardinge Medical College, New Delhi, India; b Faculty of Pharmacy, University of Medicine, Tirana, Albania; c Himalayan Institute of Medical Sciences, Swami Rama Himalayan University, Dehradun, India; d Icahn School of Medicine at Mount Sinai/Elmhurst Hospital, Elmhurst, NY, USA; e Universidad Peruana de Ciencias Aplicadas, Lima, Peru; f Universidade Estadual de Campinas (UNICAMP), Cidade Universitária “Zeferino Vaz”, Campinas, São Paulo, Brazil; g Long Island University, Brooklyn, NY, USA; h Faculty of Medicine and Surgery, University of Santo Tomas, Manila, Philippines; i Texila American University Zambia, Lusaka, Zambia; j Lviv National Medical University named after Danylo Halytskyi, Lviv, Ukraine

## Abstract

Cancer remains a leading cause of morbidity and mortality worldwide, necessitating the continued development of precision therapies that can selectively target tumor cells while minimizing off-target toxicity. Among emerging biotherapeutic strategies, monoclonal antibodies and nucleic acid aptamers (DNA and RNA) have gained prominence for their specificity and versatility in targeted cancer therapy. Antibodies are Y-shaped immunoglobulin proteins capable of recognizing tumor-associated antigens, playing a pivotal role not only in therapy but also in cancer diagnosis and detection by targeting specific tumor markers, supporting imaging modalities, and enabling immunohistochemistry; they are also the foundation of multiple approved antibody-drug conjugates. Aptamers, by contrast, are short, single-stranded DNA or RNA oligonucleotides that fold into three-dimensional structures capable of high-affinity target binding, offering advantages such as smaller molecular size, lower immunogenicity, and facile chemical synthesis. This narrative review is guided by the central question: How do antibodies and aptamers compare as platforms for targeted cancer diagnostics and therapeutics? To address this, we systematically applied the PRISMA framework to a comprehensive literature search conducted using PubMed, Scopus, and Web of Science, ensuring the systematic selection and evaluation of relevant studies published up to April 2025. Our review highlights the strengths and limitations of each biotherapeutic approach. Antibodies offer established clinical efficacy, immune effector function via Fc-mediated interactions, and favorable pharmacokinetics, but face challenges related to tumor penetration, immunogenicity, and manufacturing complexity. Aptamers, while still largely in preclinical stages, show promise due to enhanced tumor infiltration, rapid renal clearance, and engineering flexibility, though their in vivo stability and immunogenicity require further optimization. In conclusion, this review contributes a structured comparison of two major biotherapeutic platforms, emphasizing their roles not only in therapy but also in diagnosis. Both platforms hold significant clinical promise in diagnosis and therapeutics. Future research should focus on overcoming resistance mechanisms, enhancing delivery systems, and evaluating combination strategies that harness the complementary strengths of antibodies and aptamers.

## Introduction

Cancer remains one of the most formidable public health challenges worldwide, with over 20 million new cases and 9.7 million deaths reported in 2022. Projections indicate that global incidence will rise to more than 35 million new cases annually by 2050, representing a 77% increase from current levels^[^1^]^. This mounting burden highlights the urgent need for more precise and effective treatment modalities.

HIGHLIGHTS

- Antibodies and aptamers are emerging as key molecular platforms in targeted cancer diagnostics and therapy.
- Antibody-based strategies have demonstrated strong clinical success, but are limited by immunogenicity and suboptimal tumor penetration.
- Aptamers offer advantages, including improved tissue penetration, low immunogenicity, and promising translational potential.
- This review highlights the complementary strengths of antibodies and aptamers in next-generation precision oncology.
- Integrated antibody–aptamer approaches may enhance specificity, therapeutic efficacy, and diagnostic accuracy in cancer care.

Over the past two decades, the landscape of oncology has been transformed by the advent of targeted therapies, which are interventions that utilize molecular differences between malignant and normal cells^[^2^]^. Central to this strategy is biological targeting: the use of molecular agents that bind selectively to tumor-associated antigens or signaling pathways to mediate therapeutic effects^[^3^]^. Among these molecular agents are monoclonal antibodies (mAbs) and nucleic-acid aptamers (DNA and RNA). High specificity is critical not only for enhancing on-target efficacy but also for minimizing systemic toxicity and circumventing mechanisms of resistance^[^4^]^.

mAbs have emerged as the dominant modality in targeted therapy, with widespread applications in both hematological and solid malignancies. Their clinical utility spans immune checkpoint blockade, receptor antagonism, and antibody-drug conjugates (ADCs)^[^4–6^]^. Despite their successes, mAbs face limitations, including poor tumor penetration due to their large size, immunogenic potential, and high manufacturing complexity^[^7–9^]^. These challenges have spurred interest in alternative molecular formats with improved pharmacological profiles. Another molecular class is nucleic acid aptamers, which are short, single-stranded DNA or RNA oligonucleotides that represent a novel class of targeting ligands with growing relevance in oncology. Due to their smaller size, low immunogenicity, and ease of chemical modification, aptamers offer several advantages over antibodies, particularly in terms of tumor penetration, intracellular targeting, and cost-effective synthesis^[^10–12^]^. While their clinical development has been historically constrained by issues such as nuclease degradation and rapid clearance, recent advances in chemical stabilization and nanocarrier platforms have significantly enhanced their therapeutic potential^[^13,14^]^.

Targeted molecular agents suggest a potential role in tumor outcomes. However, the precise influence of these agents remains poorly understood. Guided by the framework comparing antibodies and aptamers as platforms for targeted cancer diagnostics and therapeutics, in this review we systematically conducted a comprehensive literature search and placed emphasis on the extent to which targeted molecular therapies impact therapeutic outcomes in patients with cancer. We highlight their respective mechanisms, strengths, and limitations, and discuss future directions for their integration into next-generation targeted therapies. Addressing this guiding question provides a cohesive thread that ties together existing mechanistic evidence, experimental findings, and clinical implications.

The review was prepared without the use of artificial intelligence (AI) tools in the conception, literature synthesis, drafting, or editing processes, in accordance with the TITAN 2025 guidelines^[^15^]^.

## Methodology

This review aimed to conduct a comparative analysis of antibodies and aptamers as targeted biotherapeutic strategies in oncology. A structured literature search was conducted to identify studies addressing molecular mechanisms, therapeutic applications, and translational limitations of both platforms.

The literature search was performed using major biomedical databases, including PubMed, Scopus, and Web of Science. Keywords included combinations of “monoclonal antibodies,” “aptamers,” “targeted cancer therapy,” “biotherapeutics,” “drug delivery,” and “molecular targeting.” Reference lists from relevant publications were also screened to identify additional studies pertinent to the comparative analysis.

Studies were included if they met the following criteria: (i) peer-reviewed publications discussing antibodies or aptamers in oncological applications; (ii) experimental, translational, or clinical research describing targeting mechanisms or therapeutic performance; (iii) studies evaluating diagnostic or drug-delivery strategies based on these molecules.

Articles were excluded if they focused exclusively on non-oncologic targets or lacked methodological clarity in their experimental design.

Although this work followed a structured literature identification strategy, it was designed primarily as a comparative narrative synthesis rather than a full systematic review. Consequently, the PRISMA framework was used as a guiding reference for transparency in literature selection, but quantitative meta-analysis was not performed. Evidence interpretation, therefore, relied on qualitative comparison of experimental findings, translational outcomes, and clinical development stages reported in the literature.

The biological properties of mAbs, including their specificity and immune-mediated mechanisms of action, have been extensively documented in immunotherapy research and clinical oncology studies^[^1–4^]^. By contrast, aptamers represent a relatively new class of targeting ligands, whose applications in cancer diagnostics and therapeutics have expanded significantly due to advances in nucleic acid engineering and Systematic Evolution of Ligands by Exponential Enrichment (SELEX)-based selection methods^[^5–8^]^.

## Molecular foundations of antibodies and aptamers

### Antibody structure and function

Antibodies bind to tumor-specific antigens to detect the presence of cancer cells, differentiate tumor subtypes, and assess prognosis and treatment response. There are five isotypes of antibodies – IgG, IgM, IgA, IgE, and IgD – each with distinct structures and roles in immunity. IgG is the most abundant antibody class in the blood and extracellular fluid, providing stable and lasting immunity. IgM, abundant in blood, is the first antibody produced during an immune response. IgA is found mainly at mucosal surfaces. IgE mediates responses via mast cells and basophils, collectively contributing to allergic responses and defense against parasitic infections. IgD interacts with B cells^[^16^]^.

mAbs, particularly the IgG class, are Y-shaped glycoproteins composed of two identical heavy chains and two identical light chains, forming a heterotetrameric structure. Each IgG molecule consists of two antigen-binding Fab (fragment antigen-binding) regions and one Fc (fragment crystallizable) region. The Fab domains contain variable regions that confer high specificity for antigen recognition, while the Fc region is responsible for mediating effector functions through interactions with Fc receptors and complement components^[^16,17^]^.

The therapeutic activity of IgG antibodies relies not only on antigen binding but also on immune-mediated mechanisms. Antibody-dependent cellular cytotoxicity (ADCC) is initiated when the Fc region binds to FcγRIIIa receptors on natural killer (NK) cells, resulting in the release of cytolytic granules and subsequent target cell lysis^[^18^]^. Complement-dependent cytotoxicity (CDC) occurs through Fc-mediated recruitment of the C1q component, which activates the classical complement cascade and leads to membrane attack complex formation and target cell lysis^[^19^]^. Additionally, antibodies facilitate opsonization by tagging target cells for phagocytosis through Fc receptor engagement on macrophages and neutrophils^[^20^]^.

Effector function strength varies by IgG subclass; IgG1 and IgG3 possess robust ADCC and CDC activity, whereas IgG2 and IgG4 have limited or absent effector engagement^[^17,21^]^. Glycosylation of the Fc region, especially subclass-specific N-glycan structural variations, further modulates receptor affinity and downstream immune activity^[^21,22^]^. These properties have prompted the development of engineered antibodies with optimized Fc domains to enhance or silence specific immune responses for therapeutic purposes.

Among these engineered antibody formats are single-chain variable fragments (scFvs), bispecific antibodies (bsAbs), and nanobodies that improve specificity, delivery, and function compared with traditional mAbs. Figure 1 shows these three formats. First, scFvs consist of the variable regions of the heavy and light chains of an antibody connected by a short peptide linker. They are fused with other molecules, e.g., radionuclides or effector proteins, to create ADCs for cancer recognition. On the other hand, bsAbs are built to bind two different antigens simultaneously. Combining two distinct domains into one antibody-like molecule activates specific functions such as enhanced tumor targeting, efficient tumor cell death, and reduced resistance compared with mAbs. Lastly, nanobodies are heavy-chain antibodies that are smaller yet stable, capable of penetrating solid tumors, and useful for diagnostic imaging^[^16^]^.

**Figure 1.**
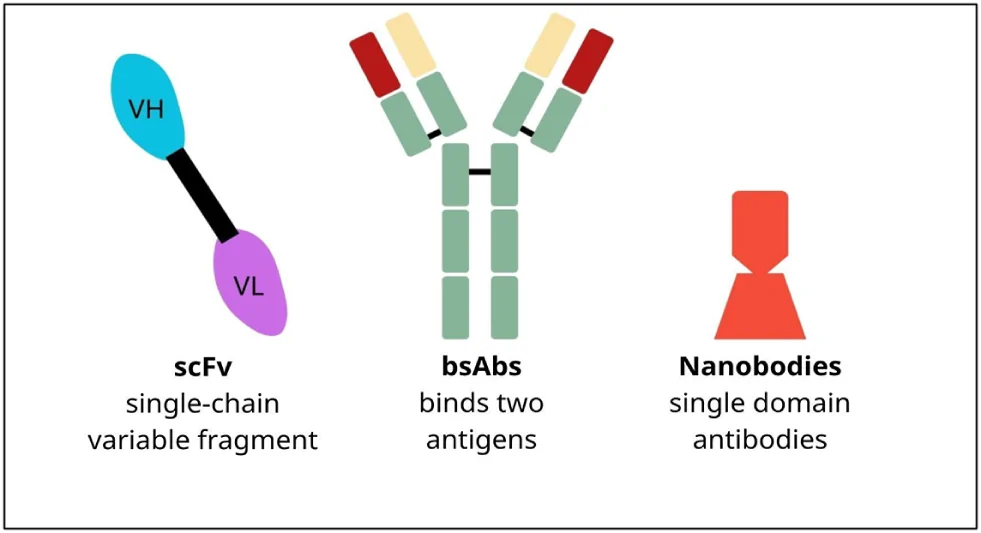
Engineered antibody formats: scFv, bsAbs, and nanobodies.

In cancer immunohistochemistry, antibodies are coupled with enzymes to detect cells of origin. There are also radiolabeled antibodies that can be used for tumor imaging to stage and detect the extent of cancer.

### Aptamer chemistry and structure

Nucleic acid aptamers are single-stranded nucleic acid molecules (either DNA or RNA) typically comprising 20–100 nucleotides. They fold into complex three-dimensional structures that enable high-affinity and high-specificity binding to molecular targets. Selected through an iterative process known as SELEX, aptamers adopt diverse secondary and tertiary motifs such as stem–loops, pseudoknots, and G-quadruplexes, creating well-defined binding surfaces via hydrogen bonding, electrostatic interactions, and van der Waals forces^[^23,24^]^.

Unlike antibodies, which are largely limited to protein targets, aptamers can recognize a broader range of molecules, including small molecules, ions, peptides, proteins, and even whole cells^[^25^]^. Their small size and synthetic origin allow access to epitopes that may be sterically inaccessible to antibodies. Moreover, aptamers can be chemically modified; for instance, with 2′-fluoro substitutions or polyethylene glycol conjugation to enhance their stability, bioavailability, and resistance to nuclease degradation^[^26^]^.

Owing to their target diversity, tunable pharmacokinetics, low immunogenicity, and scalable synthesis, aptamers offer a compelling alternative or complement to antibodies in the context of targeted cancer therapy. Figure 2 illustrates the different structures and compositions of antibodies and aptamers.

**Figure 2.**
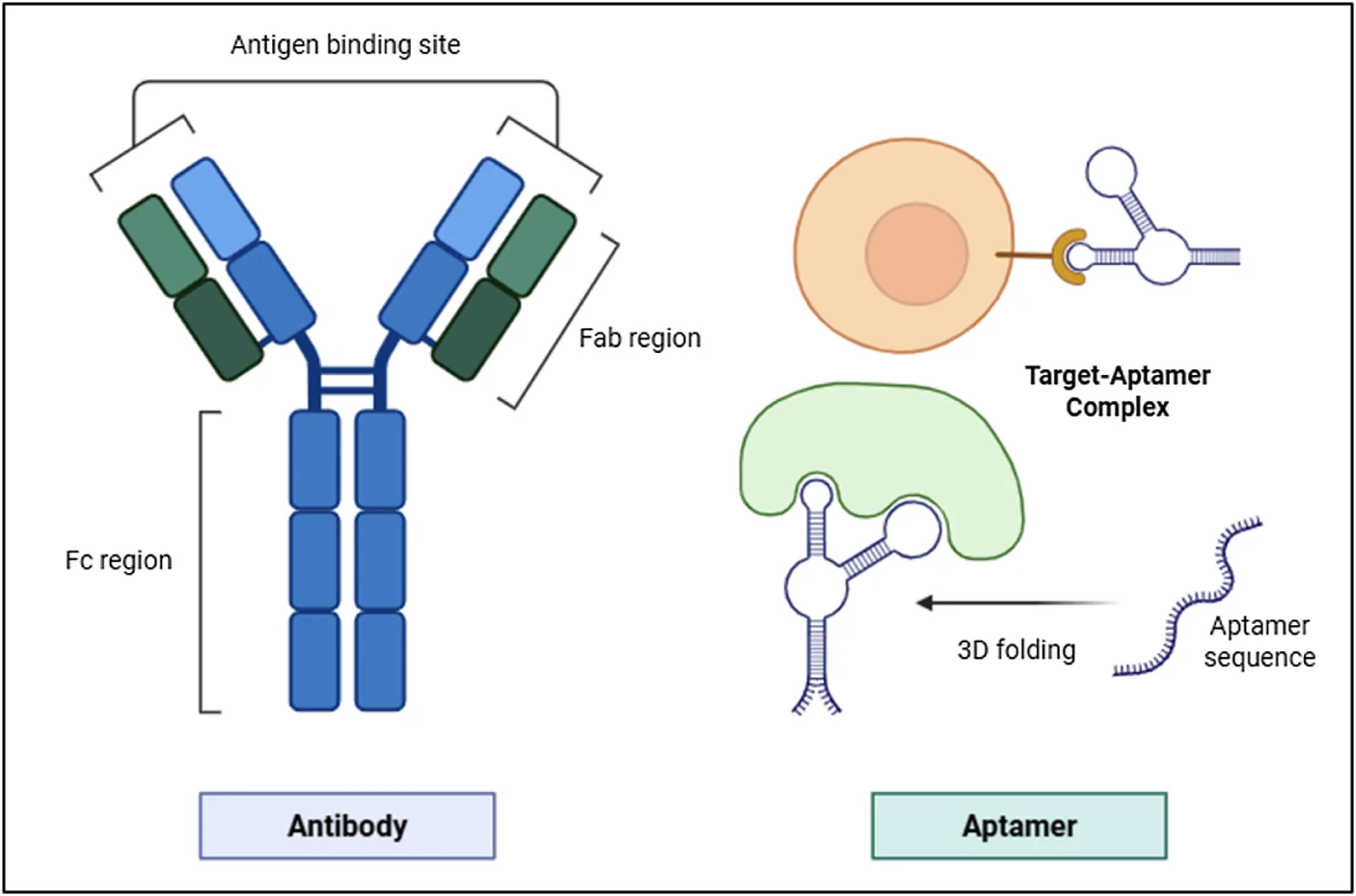
Comparative schematic of antibody vs. aptamer structures.

In cancer diagnosis, engineered aptamers can bypass multidrug resistance, extending treatment potential beyond traditional antibodies. Molecular genetic profiling, e.g., by large-scale DNA and RNA sequencing, may identify druggable molecular patterns in cancer.

## Production, engineering, and modifications

### Antibody production

#### Hybridoma production, recombinant expression, Chinese hamster ovary CHO cells, humanization, and fc engineering

The production of mAbs involves a process that integrates genetic modification with industrial process optimization to meet therapeutic and diagnostic demands. Historically, the hybridoma technique pioneered the generation of stable and specific mAbs by fusing B lymphocytes with myeloma cells to create immortalized cell lines capable of producing the desired antibodies^[^27^]^. Although effective, this methodology faces limitations related to immunogenicity and challenges associated with large-scale manufacturing.

In this context, recombinant antibody expression emerged as a solution, enabling the cloning of light- and heavy-chain genes into expression vectors that are subsequently introduced into host cells^[^28^]^. Chinese hamster ovary (CHO) cells stand out due to their ability to perform post-translational modifications that are compatible with human systems and sustain high cell densities during industrial processes^[^29^]^. Strategies such as vector design optimization, the use of synthetic promoters, and positive transcriptional feedback systems have boosted antibody yields in CHO cells, reaching levels above 5 g/L in fed-batch cultures^[^30–32**^]^**.

Recently, advances in genome engineering have enabled the direct modification of hybridoma lines, improving stability and productivity, including tools such as CRISPR/Cas9 and regulatory-region editing^[^27,33^]^. Furthermore, rapid-capture techniques, such as the use of ferrofluids to isolate human antibody-secreting cells, have allowed the generation of recombinant mAbs in shorter timeframes, enabling quicker responses to public health crises^[^34^]^.

Beyond these approaches, novel discovery platforms have reshaped antibody engineering. Phage and yeast display technologies enable high-throughput screening of extensive antibody libraries, producing binders with improved affinity and specificity^[^4,6,7^]^. In silico modeling and computational design provide predictive insights into antigen–antibody interactions, guiding affinity maturation and reducing immunogenicity risks^[^4,35,36^]^. More recently, machine learning and AI-driven platforms have accelerated discovery pipelines by analyzing sequence–function relationships, predicting developability, and optimizing antibody scaffolds^[^37–39^]^. Together, these methods shorten timelines from discovery to clinical translation while enabling highly personalized therapeutic designs.

Another crucial aspect of therapeutic antibody development is humanization and Fc region engineering. Humanization involves grafting the complementarity-determining regions (CDRs) of murine antibodies into human immunoglobulin frameworks, thereby reducing the risk of adverse immune reactions^[^34^]^. On the other hand, Fc engineering aims to adjust properties such as plasma half-life, affinity for Fcγ receptors, and the activation of effector mechanisms, including ADCC and CDC^[^35,40^]^.

Despite these advances, antibody production still encounters technical limitations and standardization barriers. Variability in glycosylation patterns, sensitivity to culture conditions, and downstream purification challenges complicate reproducibility^[^7–9^]^. Furthermore, global disparities in regulatory frameworks for quality control, biosimilarity assessment, and approval introduce additional delays. Addressing these issues requires automated bioprocessing, harmonized international standards, and multidisciplinary collaboration to ensure safety, efficacy, and accessibility.

Thus, modern antibody generation combines traditional and novel approaches, merging the precision of hybridomas with the scalability and flexibility of recombinant expression in CHO cells, alongside advanced molecular engineering techniques to maximize clinical efficacy. These innovations make antibodies increasingly adaptable therapeutic tools for personalized medicine.

### Aptamer production

#### SELEX, non-SELEX methods (microfluidics, in silico selection)

Aptamers are single-stranded DNA or RNA molecules capable of binding molecular targets with high affinity and specificity due to their well-defined secondary and tertiary structures. The primary laboratory method for their selection has been SELEX, which involves iterative cycles of binding, separation, and amplification to enrich the pool for sequences with the strongest binding characteristics^[^37,41,42^]^.

In the canonical SELEX process, an initial library containing between 10^13^ and 10^15^ variants is exposed to the target; bound sequences are separated from unbound ones and amplified via PCR or RT-PCR. Key strategies include counter-selection to eliminate ligands for unwanted epitopes, controlled variation of selection conditions (e.g., salt concentration, pH, cofactors) to ensure proper folding, and the integration of next-generation sequencing (NGS) with bioinformatic analysis to monitor enrichment and convergence of sequence families^[^36,43,44^]^.

Several SELEX adaptations have been developed to address complex targets. In cell-SELEX, whole cells are used instead of purified targets, enabling the selection of aptamers against native membrane biomarkers and allowing for the discovery of novel oncological targets; negative selection with “non-target” cell lines improves specificity^[^45,46^]^. Capture-SELEX, in turn, employs immobilization of either the library or the target on solid supports to improve separation efficiency, reduce background, and accelerate convergence, a feature particularly useful for small molecules and challenging epitopes^[^43,47^]^.

Aptamer performance can be optimized both during and after SELEX. Approaches include generating libraries biased toward desired structural motifs, adjusting the microenvironment (ions, temperature, cosolvents) to promote functional folding, structure-guided truncation to remove unnecessary regions, and chemical modifications (e.g., 2‘-F, 2’-OMe, locked nucleic acids [LNA], phosphorothioate linkages, PEGylation and/or nanoparticle conjugation) to improve nuclease resistance, affinity, and pharmacokinetics^[^41,44**,**^48^**^]^**.

More recently, microfluidic platforms have transformed SELEX by reducing reagent volumes, increasing mass transport, automating partitioning, and enabling multiplexing, which together decrease both the number of cycles and total selection time. Examples include devices for selecting aptamers against advanced glycation end-products (AGEs), which improve efficiency and reproducibility, as well as “high-dimensional” approaches that fine-tune selective affinities (K_d) via precise control of gradients and shear flows^[^49,50^]^. Studies indicate that such devices overcome the partitioning and recovery limitations of traditional SELEX and integrate effectively with NGS and in silico analysis^[^51–53^]^.

In parallel, non-SELEX methods have emerged, avoiding conventional amplification cycles. These approaches use targeted microfluidic screening to directly detect functionally relevant ligands, while in silico tools – such as molecular docking, molecular dynamics simulations, and machine learning for structure and affinity prediction, help prioritize candidates prior to laboratory validation, reducing the search space and costs^[^38,39,53,54^]^. For example, non-SELEX selection against food-derived peptides has demonstrated rapid recovery of bioactive sequences using simplified workflows^[^54^]^.

Looking ahead, hybrid pipelines combining preliminary computational screening, high-efficiency microfluidics, and minimal SELEX cycles with subsequent chemical optimization are expected to improve affinity, specificity, and stability, while reducing enrichment biases and overfitting of conformations^[^37,42,44^]^. Figure 3 illustrates the comparison between SELEX and non-SELEX techniques using a flow diagram.

**Figure 3.**
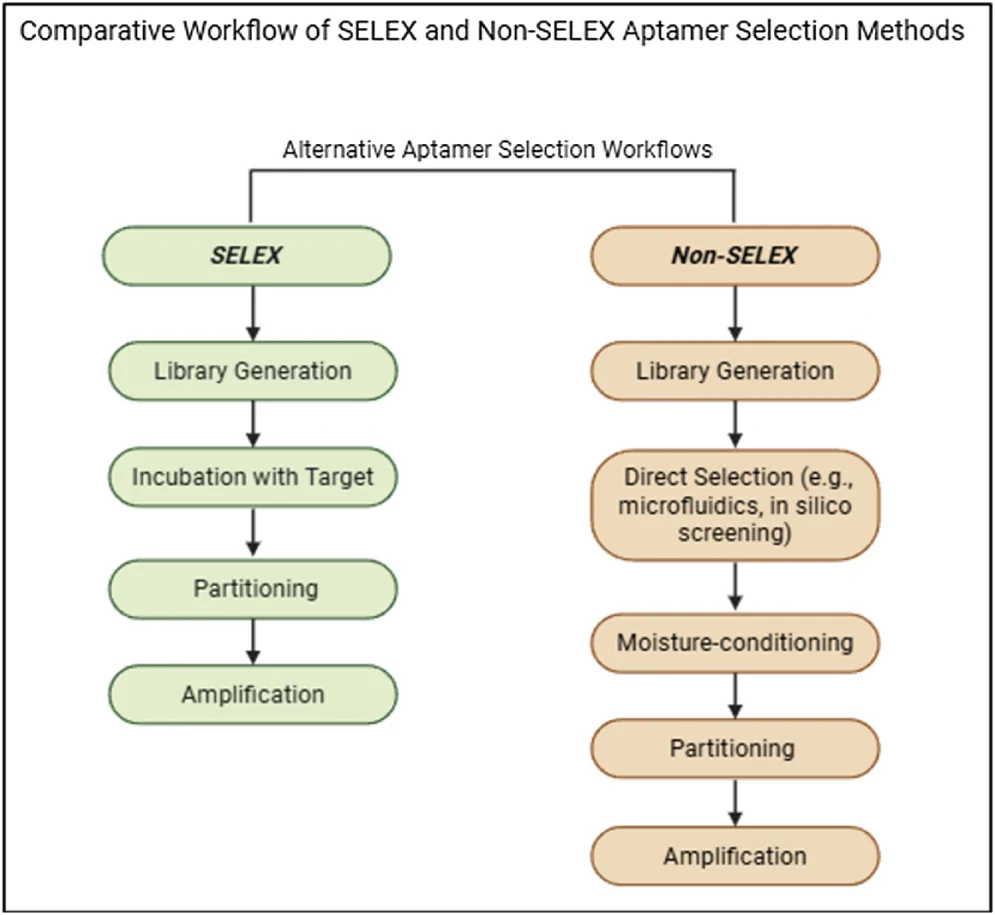
Flow diagram of SELEX vs. non-SELEX techniques.

## Applications in cancer diagnostics

Cancers induce both a pro-inflammatory and an anti-inflammatory state in the body through the activation of the humoral immune response^[^55^]^. B cells inhibit the progression of cancer by producing tumor-reactive antibodies, which may also limit their growth^[^56^]^. Isolation of immunoglobulins reactive against tumor-associated antigens has been performed in the serum as well as from the tumor milieu^[^57^]^. The presence of specific subtypes of immunoglobulins, their amount in the serum, and isotype distribution varies in patients with different cancers^[^58^]^. Thus, these antibodies provide an easy, minimally invasive means of predicting tumors, as they can be obtained from the serum^[^57^]^. Table 1 shows the application of antibodies in cancer diagnostics.

**Table 1:** shows the association of antibodies in various cancers.

Antibody	Association with cancer	Type of cancer associated	Strength/ level of association	Citation
IgA deficiency	Increased risk	Gastrointestinal cancer	IgA levels < 0.07 g/L	^[^59^]^
IgG levels	Positive association	Melanoma	<6.10 g/L	^[^60^]^
	Inverse relation	Melanoma	≥15.00 g/L	^[^60^]^
Abnormal free light chain assay	Positive association	CLL	OR = 5.56 (95% CI = 2.35–50.80, *P* = 0.0011)	^[^61^]^
IgM levels	Positive association	CLL	OR = 1.61 (95% CI 1.04–2.58, *P* = 0.034)	^[^61^]^
IgE levels	Positive association	Second squamous cell carcinoma	OR_TOTAL IGE_ = 1.44; 95% CI: 0.73–2.85	^[^62^]^
				^[^63^]^
		High-grade glioma		
	Inverse relation	Multiple myeloma	OR = 0.40 (95% CI = 0.21–0.79)	^[^64^]^
		B-cell NHL	OR = 0.62 (95% CI = 0.44–0.86)	
		CLL	OR = 0.13 (95% CI = 0.05–0.35)	
Anti EBV Ab	Inverse relation	Nasopharyngeal carcinoma	Ab vs. EBV gp350 levels	^[^65^]^
Anti RNA polymerase I and III	Positive association	Various cancers		^[^66^]^
Anti MUC 1 Ab	Inverse relation	Breast cancer		^[^67^]^
		(BRCA)		
Anti-CD25b IgG	Positive association	Lung cancer		^[^68^]^

### Aptamers in cancer diagnostics

Early cancer identification using aptamer-based probes has been explored because of their ease of modification and their ability to be truncated. These probes include several types, such as magnetic-aptamer probes, fluorescence-aptamer probes, chemiluminescence-based aptamer probes, quantum-dot-based aptasensors, colorimetric-based aptasensors, ultrasound-based aptasensors, and electrochemical-based aptasensors, among others^[^69^]^.

Contrast agents conjugated to aptamers can be used to effectively diagnose cancers radiologically^[^70^]^. In a study, CD 105-targeted aptamers were incorporated into a magnetic probe and were able to identify tumor angiogenesis^[^71^]^. In another recent study, the sgc8 aptamer-based MR contrast for MRI of lung adenocarcinoma xenografts showed increased T2-weighted signals. Aptamers can be modified with fluorescein dyes as well to generate fluorescent probes^[^72^]^. These probes have differences in efficacy depending on the type of dye used. For example, those in the bandwidth of the near-infrared emission spectrum have been shown to have better penetration in tissues and reduced autofluorescence^[^73^]^. In a study by Yang *et al*, a W5-7 aptamer was grafted with fluorescein isothiocyanate and used to detect glioma cells by fluorescence imaging in vitro^[^74^]^. The rest of the techniques are elaborated in Table 2.

**Table 2:** Shows various techniques by which aptamers can be used in cancer diagnostics.

Technique	Mechanism	Example	Citations
Chemiluminescence based aptasensor	Chemical reaction	For the detection of Romas cells	^[^75,76^]^
Quantum dots-based aptasensor	Nanoparticle based	MUC-1 aptamers for detection of C6 and Hela cells and cancer cells	^[^77^]^
Colorimetric based aptasensor	Absorption spectra analysis based	Gold nanoparticles modified with aptamers used for CCRF-CEM cells detection	^[^78^]^
Ultrasound-based aptasensor	Contrast agents decorated with aptasensors	Nanoultrasound contrast agents decorated with aptasensors in detection of prostate cancer	^[^79^]^
Electrochemical based aptasensor	Aptamers are used as recognition particles	Aptamers against MDA-MB-231 cells used for detection of circulating tumor cells	^[^80^]^

## Therapeutic applications in oncology

### Antibody therapeutics

Targeted antibody-based therapies have redefined modern oncology by offering tumor-specific interventions with improved efficacy and reduced systemic toxicity compared to conventional chemotherapy. Among these, mAbs remain one of the most versatile therapeutic modalities. Clinically approved IgG-based mAbs exert their effects through multiple mechanisms, including the direct inhibition of oncogenic signaling pathways, induction of apoptosis, and immune-mediated cytotoxicity, such as ADCC and CDC^[^5^]^. The clinical robustness of mAbs is well documented, with agents like trastuzumab, rituximab, and bevacizumab demonstrating durable benefits across breast, hematologic, and colorectal cancers, respectively. However, limitations such as resistance development, immune-related adverse events, and high treatment costs remain significant considerations for long-term management.

Recent innovations have expanded antibody therapeutics beyond conventional IgG formats. Bispecific antibodies enable dual antigen recognition, enhancing tumor selectivity and minimizing escape mechanisms, while ADCs utilize optimized linkers to deliver potent cytotoxic payloads directly to cancer cells^[^81^]^. Despite their efficacy, ADCs may induce Fc receptor–mediated immune activation and off-target toxicities, which are being addressed through improved linker chemistry, site-specific conjugation, and the development of cleavable vs. non-cleavable linker systems. Immune checkpoint inhibitors, including PD-1/PD-L1 and CTLA-4–targeting antibodies, have further revolutionized cancer therapy, particularly in melanoma and non-small cell lung cancer, by restoring anti-tumor immunity^[^82^]^.

Chimeric Antigen Receptor T-cell (CAR-T) therapy exemplifies the next frontier in antibody-derived approaches. By engineering T cells to express synthetic receptors against specific tumor antigens, CAR-T has demonstrated remarkable efficacy in hematologic malignancies such as acute lymphoblastic leukemia (ALL) and diffuse large B-cell lymphoma (DLBCL)^[^83^]^. Ongoing refinements include armored CARs, switchable constructs for safety modulation, and exploration of solid tumor–associated antigens^[^84^]^. Nevertheless, challenges such as cytokine release syndrome, neurotoxicity, and antigen escape underscore the need for improved strategies in both antibody- and CAR-T–based modalities.

The therapeutic mechanisms of antibody-based platforms – including effector recruitment, targeted payload delivery, and checkpoint inhibition – contrast with nucleic acid–based strategies such as aptamers, which rely primarily on molecular recognition and synthetic modification for tumor targeting (Fig. 4).

**Figure 4.**
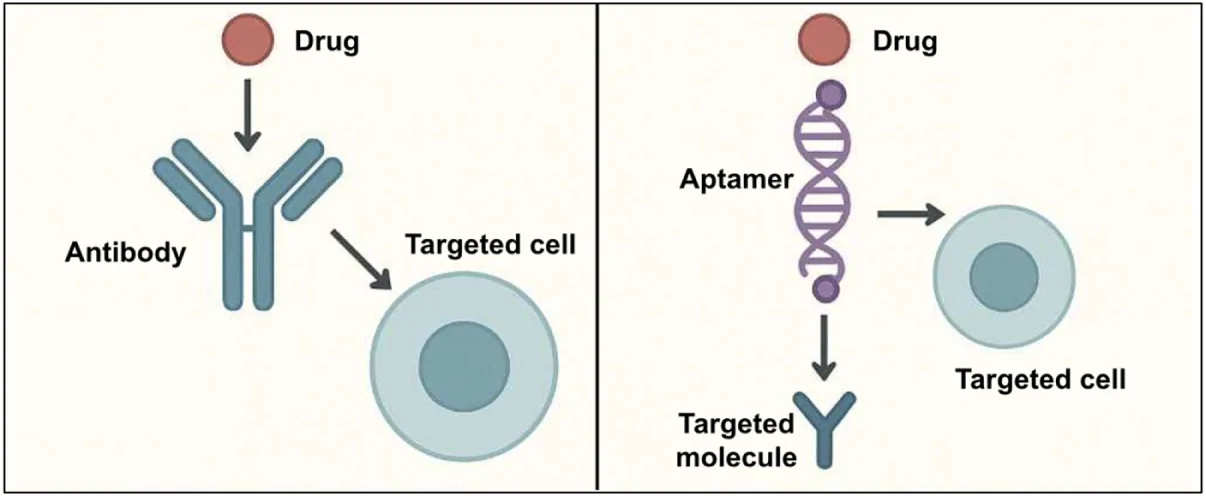
Mechanisms of targeted delivery using antibodies vs. aptamers.

### Aptamer-based therapeutics

Aptamers represent a rapidly advancing class of nucleic acid–based therapeutics with promising applications in oncology. Structurally distinct from protein-based antibodies, aptamers are short single-stranded oligonucleotides capable of folding into complex three-dimensional conformations that bind targets with high specificity and affinity. Their small size, low immunogenicity, rapid tissue penetration, and cost-effective synthesis offer unique translational advantages^[^85^]^. Unlike antibodies, aptamers can be readily modified with chemical moieties to enhance nuclease resistance, extend half-life, or incorporate imaging agents and cytotoxic drugs.

Among the most clinically relevant applications are aptamer-drug conjugates (ApDCs), where aptamers function as precision-guided delivery vehicles for chemotherapeutics or nucleic acids. For example, prostate-specific membrane antigen (PSMA)-binding aptamers have shown robust preclinical efficacy in prostate cancer by selectively delivering cytotoxic payloads or siRNAs to tumor cells, thereby reducing systemic toxicity^[^86,87^]^. Nanotechnology platforms – such as liposome- or nanoparticle-based carriers functionalized with aptamers – are being explored in preclinical and early clinical settings to optimize drug stability, enhance biodistribution, and achieve controlled release kinetics^[^88^]^. These systems exemplify how aptamer engineering can overcome challenges of rapid clearance and improve therapeutic outcomes (Fig. 4).

Nevertheless, limitations persist, including susceptibility to renal clearance and potential loss of function in the tumor microenvironment. Strategies under investigation to address these challenges include PEGylation, LNA incorporation, and multivalent aptamer designs. Furthermore, while aptamers are generally considered less immunogenic than protein therapeutics, quantitative data comparing immune activation across modalities are limited. Reduced immunogenicity implies greater feasibility for repeated dosing, but rigorous comparative studies are required to validate this claim and establish dosing regimens compatible with long-term use.

Collectively, antibody- and aptamer-based therapeutics represent complementary strategies in precision oncology. Antibodies provide robust clinical outcomes with diverse effector functions, while aptamers offer unique pharmacologic and manufacturing advantages that may enable broader accessibility and personalization of cancer therapies.

## Regulatory landscape and clinical translation

The regulatory trajectory of antibody-based therapeutics differs significantly from that of aptamer-based drugs. mAbs have become well-established in oncology, with numerous agents receiving regulatory approval for the treatment of hematologic malignancies and solid tumors. Their clinical success is largely attributed to their high specificity for tumor-associated antigens and their ability to recruit immune effector mechanisms^[^1,2^]^.

More recently, antibody-based platforms such as antibody–drug conjugates (ADCs) have expanded the therapeutic potential of antibody targeting by enabling the selective delivery of cytotoxic payloads to tumor cells^[^9,10^]^. These conjugates have demonstrated significant clinical efficacy but also present challenges related to toxicity, resistance mechanisms, and pharmacokinetic variability^[^11–13^]^.

In contrast, aptamer-based therapeutics remain underrepresented in clinical oncology despite promising preclinical results. Aptamers offer several theoretical advantages, including low immunogenicity, high binding specificity, and chemical synthesis scalability^[^5,6**^]^** However, their translation into clinical practice has been limited by pharmacokinetic instability, rapid renal clearance, and susceptibility to nuclease degradation^[^14–16^]^.

These challenges highlight the importance of continued development of chemically modified aptamers, nanoparticle-based delivery systems, and hybrid targeting platforms designed to improve stability and therapeutic efficacy^[^17–20^]^.

## Drug delivery and immunomodulation

The clinical success of targeted cancer therapy depends not only on binding specificity but also on effective drug delivery and immune modulation. ADCs exemplify this dual functionality, offering the ability to selectively deliver cytotoxic agents to tumor cells while also engaging the immune system^[^89^]^. ADCs benefit from the inherent stability of the antibody scaffold, allowing for prolonged circulation and protection of the payload from premature degradation^[^90^]^. However, ADC efficacy is often limited by resistance mechanisms of the tumor, including antigen downregulation, impaired internalization, dysfunctional lysosomal processing, drug efflux, and payload-related resistance^[^91,92^]^. Moreover, certain ADCs can elicit immune-related adverse events due to Fc receptor-mediated immune activation or off-target effects, necessitating careful linker and payload design to balance efficacy and safety^[^93,94^]^.

In contrast, aptamer-drug conjugates (ApDCs), particularly when engineered as part of nanoparticle-based delivery systems, present promising alternatives. Aptamers are short, single-stranded oligonucleotides that can be synthesized and modified with high precision^[^95^]^. Their smaller size enables deeper tumor infiltration and more homogeneous distribution within the tumor microenvironment^[^96^]^. Aptamer-based nanocarriers also offer modularity, allowing for the incorporation of multiple therapeutic agents or imaging probes^[^97^]^. Despite these advantages, aptamers face challenges related to in vivo stability, susceptibility to nuclease degradation, and rapid renal excretion^[^98,99^]^. Chemical modifications and encapsulation strategies have improved their pharmacokinetics, yet concerns about immunogenicity persist^[^99^]^. While generally considered less immunogenic than protein-based therapeutics, certain sequences or delivery platforms can still provoke unintended immune responses^[^100^]^.

Another key difference lies in immunomodulatory potential. ADCs, through Fc-mediated interactions, can recruit immune effector cells such as NK cells and macrophages, potentially enhancing antitumor immunity^[^101^]^. Aptamers lack this Fc region, limiting direct immune activation, though this can be addressed through combination strategies or aptamer-functionalized immunotherapeutics. Ultimately, the choice between ADCs and ApDCs hinges on the therapeutic context, desired pharmacologic profile, and the tumor’s immune landscape (Table 3).

**Table 3:** Comparative analysis of antibody-drug conjugates (ADCs) and aptamer-drug conjugates (ApDCs) in drug delivery and immunomodulation^[^96,99,100^]^.

	Antibody-drug conjugates (ADCs)	Aptamer–drug conjugates (ApDCs)
Molecular size	∿150 kDa	∿30 kDa
Tumor penetration	Limited due to its large size	High/enhanced
Stability	Susceptible to changes in temperature and pH	High, improved with modifications
Immunogenicity	Unwanted immunogenicity	Minimal
Clearance	Slower, prolonged half-life	Rapid renal excretion
Manufacturing costs	High, requires complex biological production	Lower, scalable chemical synthesis

## Failure analysis of aptamer clinical development

Despite strong performance in vitro and in preclinical models, many aptamer candidates have faced challenges during clinical development. One major limitation is the susceptibility of unmodified nucleic acid aptamers to enzymatic degradation, which significantly reduces their circulating half-life^[^14,15**^]^**. Another limitation involves rapid renal filtration due to the relatively small molecular weight of aptamers compared with antibodies. This can reduce effective tumor exposure unless chemical modifications or carrier systems are introduced^[^16–18^]^.

In addition, differences between controlled in vitro conditions and the complex biological environment encountered in vivo may alter aptamer binding behavior, leading to reduced target affinity and therapeutic efficacy^[^19,20^]^. Recent studies therefore focus on structural engineering strategies, including chemical backbone modification, PEGylation, and aptamer–nanoparticle conjugation, which aim to improve pharmacokinetic stability and tumor targeting^[^21–24^]^.

## Future challenges and innovations

Antibodies possess superior circulatory stability due to their larger molecular size, organized protein domains, and Fc-mediated recycling mechanisms^[^11^]^. In contrast, aptamers present multiple critical limitations relative to antibodies, most notably in terms of in vivo stability and pharmacokinetics. Their small molecular size, while beneficial for deep penetration into solid tumors and the tumor microenvironment, simultaneously promotes vulnerability to nuclease-mediated degradation and rapid renal clearance, frequently yielding plasma half-lives of under 2 hours. This creates a significant obstacle in achieving both efficient tumor targeting and sufficient systemic retention^[^11,102–104^]^. Another challenge is the limited availability of comprehensive human safety data, which necessitates vigilant evaluation of unintended immune responses, particularly when chemical modifications are introduced^[^11,102,105^]^. From a manufacturing perspective, regulatory pathways for oligonucleotide-based therapies – especially from agencies such as the FDA and EMA – remain less mature and standardized for aptamers than those applied to mAbs^[^11,103,106^]^. Although the SELEX process enables the identification of high-affinity aptamers, it involves a laborious, iterative screening process and can result in molecules with suboptimal serum stability or off-target binding, which impairs their translational potential^[^102,103,105^]^.

Current innovations are tackling these challenges through sophisticated chemical modifications, including 2’-O-methyl, 2’-fluoro substitutions, LNAs, PEGylation, and circularization. These strategies significantly improve resistance to nucleases and prolong systemic circulation time^[^102,103,105^]^. Additionally, aptamers are being incorporated into multifunctional platforms such as nanomaterials, DNA origami structures, and hybrid constructs. These platforms enable targeted drug delivery, siRNA co-administration, and combination therapies, which are modalities often constrained by the larger size and complexity of antibody-based systems^[^107^]^. Importantly, aptamers can be engineered to counteract multidrug resistance (MDR) mechanisms, targeting proteins like P-glycoprotein or Bcl-2, thereby offering therapeutic avenues where traditional antibodies fall short^[^104^]^. Their modular and fully synthetic nature supports rapid, patient-specific aptamer design, facilitating adaptive strategies in precision oncology for evolving tumor antigens and neoantigens^[^102,103,106^]^.

In hematologic malignancies, integrating CRISPR and riboswitches with aptamer-based systems represents an emerging therapeutic approach. CRISPR is a gene-editing technology that regulates gene expression in target leukemic cells, thereby improving specificity^[^108^]^. Use of this modality has the potential to improve recognition of target tumors and to facilitate the study of target genes essential for tumor survival.

In conclusion, aptamers hold considerable potential for targeted cancer therapy due to their specificity, synthetic flexibility, and superior tumor penetration. Nonetheless, bridging the gap between laboratory success and clinical application will require robust preclinical validation and harmonized regulatory standards.

## Comparative evaluation: quantitative and translational aspects

Antibodies and aptamers share the ability to recognize molecular targets with high specificity, yet their physicochemical properties and translational characteristics differ substantially.

mAbs typically exhibit high binding affinity and prolonged systemic half-life due to their large molecular structure and Fc-mediated recycling pathways^[^1,2^]^. These features contribute to their widespread use in clinical oncology, but also increase production complexity and cost because antibodies require mammalian-cell expression systems and extensive purification processes.

In contrast, aptamers are chemically synthesized nucleic acid ligands that can be produced with high reproducibility and scalability^[^5,6^]^. Their smaller molecular size enables rapid tissue penetration, which may be advantageous for targeting certain tumor microenvironments^[^19,21^]^.

However, aptamers also exhibit rapid systemic clearance and reduced *in vivo* stability unless chemically modified^[^14–16^]^. Consequently, several strategies have been developed to enhance their therapeutic potential, including aptamer–drug conjugates, aptamer-functionalized nanoparticles, and hybrid targeting constructs^[^17,22–25^]^.

## Risk-of-bias considerations

The studies analyzed in this review include preclinical experiments, translational research, and clinical investigations. Potential sources of bias arise from heterogeneity in tumor models, variability in experimental design, and differences in methodological reporting across studies.

Preclinical research often relies on controlled cellular or animal models, which may overestimate translational potential. In contrast, clinical investigations provide stronger evidence regarding safety and efficacy but remain limited in number for aptamer-based therapeutics compared with antibody-based drugs^[^1,5,14^]^.

These factors should be considered when interpreting comparative findings between antibodies and aptamers in oncology.

## Limitations of the present review

This study presents several limitations. First, although a structured literature identification strategy was applied, the review was not designed as a full systematic review with meta-analysis. Consequently, a quantitative synthesis of outcomes across studies was not performed.

Second, heterogeneity in experimental models, tumor types, and therapeutic endpoints limited the ability to perform direct quantitative comparisons between antibodies and aptamers.

Finally, the relatively small number of clinical trials investigating aptamer-based therapeutics limits the strength of conclusions regarding their translational potential in oncology^[^14,19,21^]^.

## Future research framework

Future research should focus on several key areas that may enhance the translational potential of aptamer-based therapeutics.

First, the development of chemically stabilized aptamers capable of resisting nuclease degradation and extending systemic half-life remains a major priority^[^15,16^]^. Second, hybrid therapeutic platforms combining aptamers with nanoparticles, antibodies, or drug conjugates may improve tumor targeting and drug delivery efficiency^[^17,22,23^]^.

Additionally, standardized comparative preclinical models should be developed to allow more rigorous evaluation of targeting performance across different biotherapeutic platforms.

Advances in molecular engineering, nanotechnology, and precision medicine may ultimately allow aptamers to complement antibody-based therapeutics as part of integrated targeted treatment strategies in oncology^[^24–26^]^.

## Conclusion

Cancer remains a global health priority, with rising incidence demanding more precise and accessible therapies. Within this framework, antibodies and aptamers emerge as complementary biotherapeutic platforms. Antibodies have achieved remarkable clinical success, driven by advances in hybridoma technology, recombinant expression in CHO cells, and engineering strategies such as Fc modification. More recently, discovery pipelines incorporating phage and yeast display, computational modeling, and machine learning–assisted optimization have accelerated antibody development and improved therapeutic design. Despite these strengths, challenges remain, including immunogenicity, high manufacturing costs, and the need for international standardization to ensure reproducibility and regulatory alignment. In parallel, aptamers offer distinct advantages, including small molecular size, low immunogenicity, and ease of chemical synthesis. Their modularity and ability to penetrate tumors make them particularly promising for drug delivery and combination strategies. Although limited by rapid clearance and stability issues, innovations in SELEX adaptations, chemical modifications, and microfluidic platforms are progressively overcoming these barriers. Future strategies should integrate the immune effector capabilities of antibodies with the engineering flexibility of aptamers. Comparative clinical studies and harmonized regulatory frameworks will be essential to translate these advances into practice. Together, these platforms hold the potential to reshape precision oncology in the face of the escalating global cancer burden.

## Data Availability

No new datasets were generated or analyzed during the preparation of this review article. All data discussed are derived from previously published studies and have been appropriately cited.

## Research registration unique identification number (UIN)

Not applicable.

This study is a narrative review and does not involve clinical trials, patient recruitment, or experimental interventions.
